# Yellow Fever in Buenos Aires

**DOI:** 10.3201/eid3202.AC3202

**Published:** 2026-02

**Authors:** Terence Chorba

**Affiliations:** Centers for Disease Control and Prevention, Atlanta, Georgia, USA

**Keywords:** yellow fever, vector-borne infections, Juan Manuel Blanes, An episode of yellow fever in Buenos Aires, art–science connection

**Figure 1:**
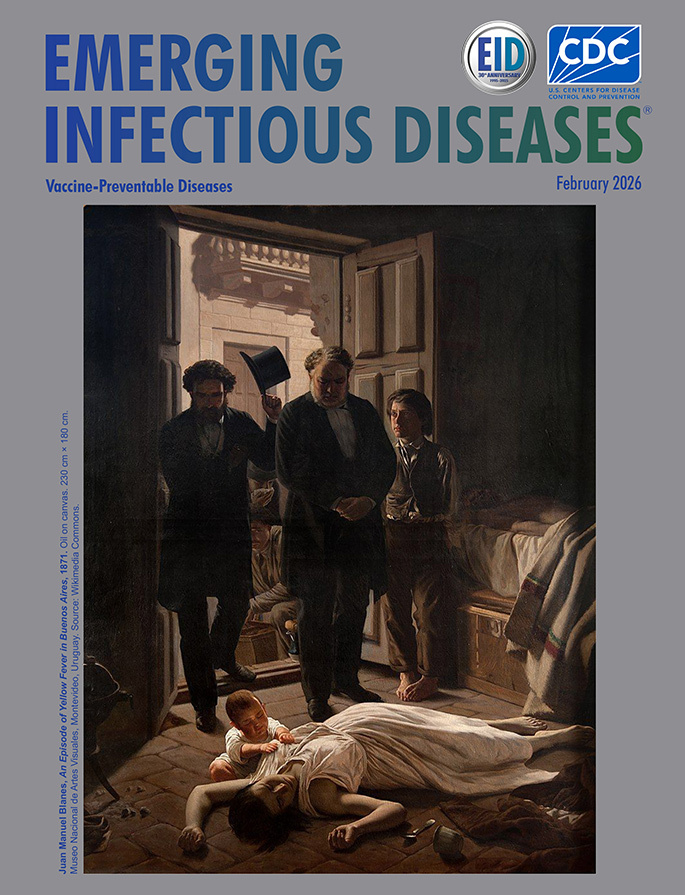
**Juan Manuel Blanes, *An Episode of Yellow Fever in Buenos Aires*, 1871.** Oil on canvas. 230 cm × 180 cm. Museo Nacional de Artes Visuales, Montevideo, Uruguay. Source: Wikimedia Commons.

A vectorborne disease is an illness caused by viruses, bacteria, parasites, or fungi transmitted by arthropod vectors such as mosquitoes, ticks, or fleas. Annually, vectorborne diseases account for >700,000 deaths globally, including deaths from malaria, dengue, schistosomiasis, Chagas disease, yellow fever, human African trypanosomiasis, leishmaniasis, Japanese encephalitis, and onchocerciasis (*1*). Yellow fever is a viral disorder caused by an *Orthoflavivirus* that is a genus in the Flaviviridae family and is so named after the jaundice (yellowing of the eyes and skin) that is associated with yellow fever virus infection; flavus means yellow in Latin. All orthoflaviruses are small, spherical, enveloped viruses, ≈50 nm in diameter, sharing a similar architecture consisting of an ≈11,000-base single-stranded RNA virus genome, a nucleocapsid of a protein shell containing the viral RNA, and a host-derived lipid envelope that surrounds the nucleocapsid (*2*,*3*).

Yellow fever virus is transmitted to humans primarily through the bite of infected mosquitoes. The virus replicates in the liver and other organs, causing overwhelming inflammation and damage. Infection with this virus is believed to have first appeared in the Americas through the transatlantic slave trade from Africa, causing repeated epidemics in the 18th and 19th Centuries, primarily in the port cities of both North and South America. The disease often caused widespread terror and economic disruption, and notable outbreaks occurred in Philadelphia (1793), New Orleans (1853), and Buenos Aires (1871); lesser outbreaks had been reported in Buenos Aires in 1852, 1858, and 1870. It is now known that most orthoflaviviruses, including yellow fever virus, cause subclinical infections that go undetected in existing clinical-based disease surveillance programs (*4*); >85% of yellow fever virus infection cases are either asymptomatic or result in only mild illness (*5*). To this day, yellow fever remains endemic and is widely distributed in the tropical areas of Africa and Latin America, reportedly accounting for 67,000–173,000 severe infections and 31,000–82,000 deaths annually (*6*,*7*).

The scene featured on this month’s cover, *An Episode of Yellow Fever in Buenos Aires* (*Un Episodio de la Fiebre Amarilla en Buenos Aires*), painted by Uruguayan artist Juan Manuel Blanes in 1871, is a depiction of the tragic events of death in a family from the yellow fever epidemic that devastated Buenos Aires in that same year. It is a somewhat chiaroscuro composition—that is, there is great contrast between light and darkness, although it is predominantly dark with ochres and grays. In his work, Blanes depicts the images of a man, lying dead on a bed and obscured in darkness, and of a young woman sprawled on a stone floor, also dead from the disease. Their orphaned child is seated beside the mother in stunned desperation, struggling to access her breast. Behind them are 2 members from the city’s People’s Commission (Comisión Popular) who have come upon the scene, lawyer José Roque Pérez (center) and Dr. Manuel Argerich (to his right), both of whom later also died in the epidemic as the result of their dedicated work, supporting the sick (*8*). The same epidemic lay claim to a reported 14,000 lives in an estimated population of 180,000; among them was another great artist and colleague of Blanes, the Argentine American Franklin Rawson (1791–1871), who was renowned for genre scenes and historical paintings and portraits with elements of romantic realism very similar to those in the work of Blanes (Figure 1) (*9*).

**Figure 2:**
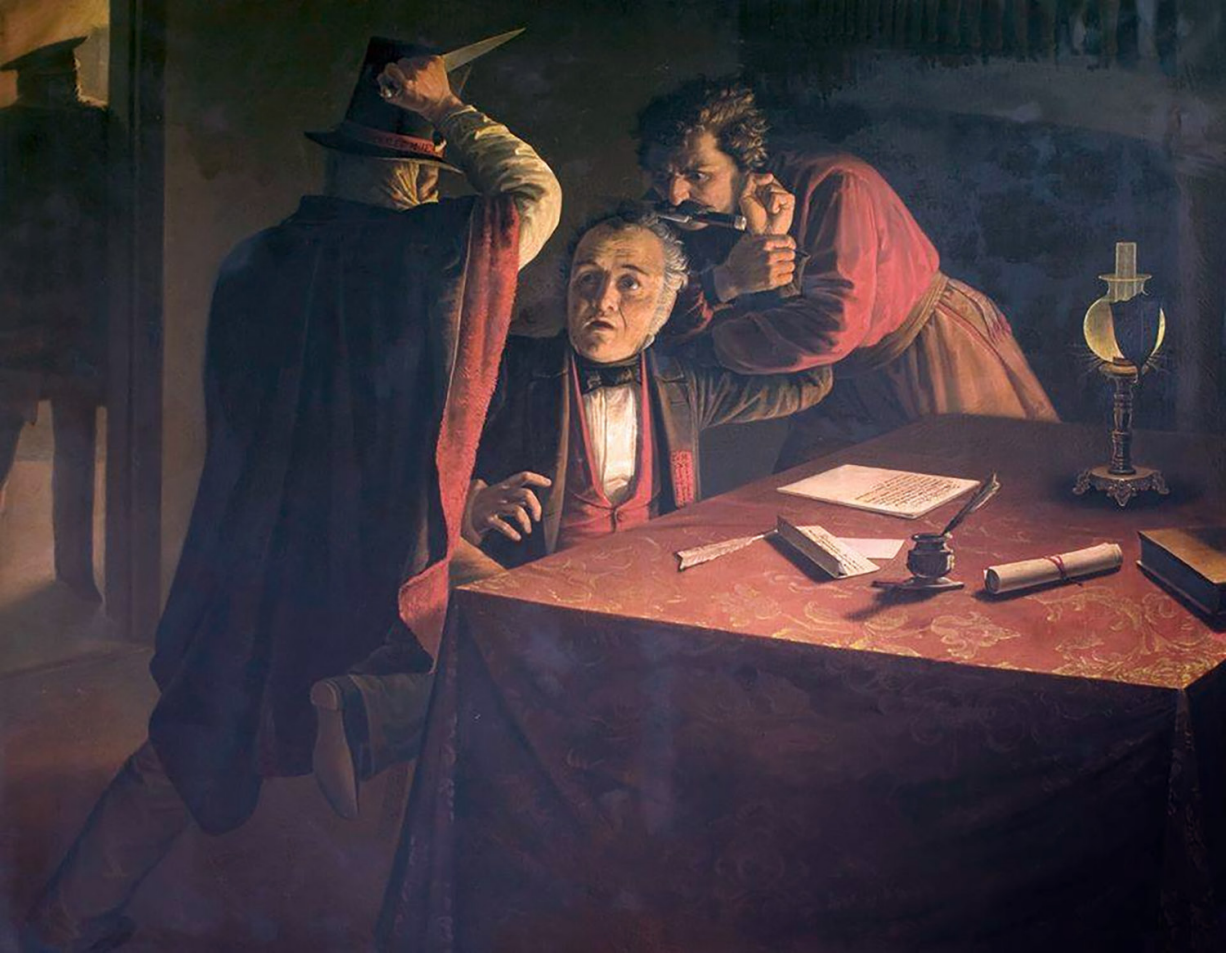
Franklin Rawson, *Murder of Manuel Vicente Maza*, 1860. Oil on canvas. 175 cm × 219.5 cm. Complejo Museográfico Provincial “Enrique Udaondo,” Ciudad de Luján, Buenos Aires, Argentina. Source: Wikimedia Commons.

Juan Manuel Blanes was born in Montevideo, Uruguay, a newly independent country, in 1830, and from an early age demonstrated great talent serving as a self-taught illustrator and portrait painter in a politically destabilized environment—Uruguay endured a civil war from 1843 to 1851. In 1860, Blanes obtained a scholarship from the Uruguay government, and for several years, he studied under Antonio Ciseri, a widely recognized Italian portrait and neoclassicist realism painter of religious works (*10*). The influence of Ciseri can be seen in the photographic depiction of the family lost to disease in the Buenos Aires epidemic, Blanes’ earliest renowned work. Blanes subsequently had a very successful and prolific career in Uruguay, Chile, Argentina, and Italy, making historical paintings, portraits, and depictions of gauchos (nomadic horsemen and cowhands of the grasslands in Uruguay and Argentina, renowned in the mid-18th to mid-19th Century) (Figure 2), and died in Pisa, Italy, in 1901, at the age of 70 (*11*). In recognition of his talent and contribution to his nation, the city of Montevideo named its Juan Manuel Blanes Municipal Museum of Fine Arts in his honor in 1930.

**Figure 3:**
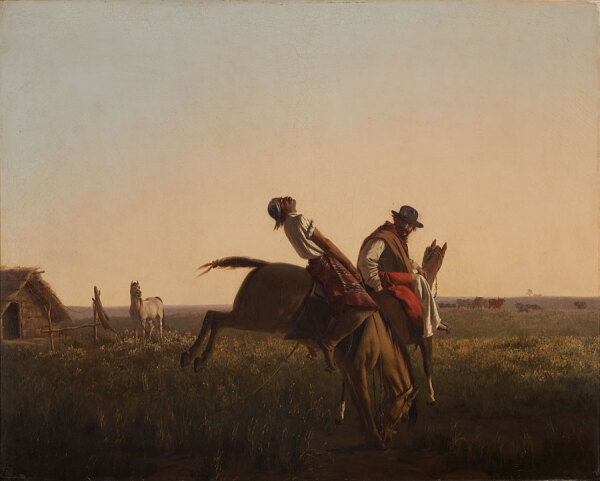
Juan Manuel Blanes, The Dressage, ca. 1875. Oil on canvas. 80 cm × 100 cm. Museo Nacional de Artes Visuales, Montevideo, Uruguay. Source: Wikimedia Commons. Photograph by Eduardo Baldizan.

The first experiments that demonstrated that mosquitoes accounted for yellow fever transmission were conducted by Walter Reed and his colleagues at the turn into the 20th Century, building on findings of Carlos Finlay, a Cuban physician, who first theorized the transmission problem in 1881 (*12*). The history of yellow fever took another favorable turn in the 20th Century with the development of a yellow fever vaccine, for which Max Theiler was recognized with a Nobel Prize in 1951 (*13*). Fortunately, the vaccine has saved many lives, and investment in its development has resulted in a marked diminution in the frequency of scenarios like that depicted in the Blanes’ yellow fever painting, by preventing or interrupting the outbreaks that repeatedly would devastate so many communities (*12*).
